# Systematic scoping review protocol of methodologies of chronic respiratory disease surveys in low/middle-income countries

**DOI:** 10.1038/s41533-019-0129-7

**Published:** 2019-05-08

**Authors:** Dhiraj Agarwal, Nik Sherina Hanafi, Soumya Chippagiri, Evelyn A. Brakema, Hilary Pinnock, Ee Ming Khoo, Aziz Sheikh, Su-May Liew, Chiu-Wan Ng, Rita Isaac, Karuthan Chinna, Wong Li Ping, Norita Binti Hussein, Sanjay Juvekar, the RESPIRE Collaborators, D. Das, B. Paul, H. Campbell, E. Grant, M. Fletcher, S. Saha, M. Habib, S. El Arifeen, R. Huque, P. Khatavkar, S. Salvi, S. Yusuf, M. O. Yusuf, N. Bashir

**Affiliations:** 1 Vadu Rural Health Program, KEM Hospital Research Centre, Pune, India; 2 Department of Primary Care Medicine, Faculty of Medicine, University of Malaya, Kuala Lumpur, Malaysia; 3 RUHSA Department, Christian Medical College, Vellore, India; 4 Department of Public Health and Primary care, Leiden University Medical Centre, Leiden, The Netherlands; 5 NIHR Global Health Research Unit on Respiratory Health (RESPIRE), Usher Institute of Population Health Sciences and Informatics, The University of Edinburgh, Edinburgh, UK; 6 Child Health Research Foundation, Dhaka, Bangladesh; 7 Bangladesh Primary Care Respiratory Society, Dhaka, Bangladesh; 8 Maternal and Child Health Division (MCHD), International Centre for Diarrhoeal Disease Research, Dhaka, Bangladesh; 9 University of Dhaka, Dhaka, Bangladesh; 10 KEM Hospital, Pune, India; 11 Chest Research Foundation, Pune, India; 12 The Allergy & Asthma Institute, Islamabad, Pakistan

**Keywords:** Chronic obstructive pulmonary disease, Asthma

## Abstract

This protocol describes a systematic scoping review of chronic respiratory disease surveys in low/middle-income countries (LMICs) undertaken as part of the Four Country ChrOnic Respiratory Disease (4CCORD) study within the National Institute for Health Research Global Health Research Unit on Respiratory Health (RESPIRE). Understanding the prevalence and burden of chronic respiratory disease (CRD) underpins healthcare planning. We will systematically scope the literature to identify existing strategies (definitions/questionnaires/diagnostics/outcomes) used in surveys of CRDs in adults in low-resource settings. We will search MEDLINE, EMBASE, ISI WoS, Global Health and WHO Global Health Library [search terms: prevalence AND CRD (COPD, asthma) AND LMICs, from 1995], and two reviewers will independently extract data from selected studies onto a piloted customised data extraction form. We will convene a workshop of the multidisciplinary 4CCORD research team with representatives from the RESPIRE partners (Bangladesh, India, Malaysia, Pakistan and Edinburgh) at which the findings of the scoping review will be presented, discussed and interpreted. The findings will inform a future RESPIRE 4CCORD study, which will estimate CRD burden in adults in Asian LMICs.

## Background

Chronic respiratory diseases (CRDs), especially asthma and chronic obstructive pulmonary disease (COPD), are common public health problems across the world, with the Global Burden of Disease estimating that CRDs now account for 30% of total deaths.^1^ Although morbidity and mortality are particularly high in low- and middle-income countries (LMICs), there are very little robust data on the true prevalence of asthma and COPD in these countries.^2,3^ Chronic respiratory symptoms are common in the general population,^1^ but the clinicians in primary healthcare systems in resource-poor countries lack the skills and support to diagnose the underlying disease condition.^4–8^ Factors contributing to low rates of diagnosis include limited awareness of respiratory long-term conditions,^4–10^ limited access to healthcare and lack of diagnostic capability in these countries.^11^ Determining the prevalence of asthma and COPD remains a challenge because of the poor sensitivity and specificity of the widely used questionnaire-based research tools,^3,12^ while objective testing with spirometry may be a challenge in community-based epidemiological surveys.^11^

With notable exceptions, such as the Burden of Lung Disease (BOLD),^13^ surveys of the prevalence of CRDs conducted in LMICs often have major limitations (such as relying on patient-reported disease or symptom questionnaires), and report very varied estimates of prevalence.^2,14–39^ In addition, many existing surveys focus on one condition (e.g. the BOLD study detects COPD,^13^ the International Study of Asthma and Allergies in Childhood (ISAAC) detects symptoms of asthma and allergy in children^40^) and rarely look for the broad range of less common causes of CRD (such as interstitial lung disease, bronchiectasis, lung cancer and complications post tuberculosis) or attempt to identify the phenotypes of asthma and COPD, which are increasingly regarded as important to understanding and managing the conditions.^41^

Effective health policies related to CRD can only be developed if we know the true burden of asthma, COPD and other CRDs in the community. Funded by the National Institute for Health Research (NIHR), RESPIRE is a Global Health Research Unit focusing on respiratory health in Asia (https://www.ed.ac.uk/usher/respire). Prior to undertaking a comprehensive Four Country ChrOnic Respiratory Disease study (4CCORD) in the partner countries of RESPIRE, we sought to systematically scope the literature to identify existing strategies—that is, definitions, questionnaires, study tools and diagnostics protocols—that have been used to conduct surveys for CRDs in LMICs.

## Methods

A systematic scoping review aims to ‘map the key concepts underpinning a research area and the main sources and types of evidence available’.^42^ As such, scoping reviews typically address broad questions, potentially include a range of methodologies and do not undertake quality assessment. This contrasts with the focused question of a systematic review, which is answered from a relatively narrow range of quality-assessed studies.^42^

Systematic review procedures, as described in the Cochrane Handbook for Systematic Reviews of Interventions,^43^ have been adapted to meet the needs of a scoping review.^44,45^ We will follow the six-step framework described by Arksey and O’Malley^42^ and subsequently widely endorsed and enhanced in methodological literature.^45–47^

### Identifying the research question

The aim of this review is to scope published epidemiological CRD surveys to identify their aims, methodologies and outcomes in order to identify evidence gaps and inform the methodology of the proposed RESPIRE 4CCORD survey on the burden of CRD. Our objectives are therefore to answer the following research questions:

1. What surveys on the prevalence of asthma, COPD and other CRDs have been undertaken in resource-poor settings?
2. What definitions, questionnaires, tests, diagnostic processes and outcomes did the surveys employ?
3. How was the socio-economic burden (from a societal or healthcare perspective) of asthma, COPD and other CRDs estimated in these surveys?
4. What strategies have been used to identify phenotypes of asthma and COPD, or to identify the causes of ‘other CRD’?

### Identifying relevant studies

We will search MEDLINE, EMBASE, ISI Web of Science, Global Health and WHO Global Health Library using search terms for prevalence AND CRD (COPD OR asthma OR CRD) AND LMICs/low-resource settings, from year 1995 onwards when publication of the Global Initiative for Asthma (GINA),^48^ followed by the Global Initiative for Chronic Obstructive Lung Disease (GOLD) guidelines for COPD,^49^ provided internationally agreed definitions and diagnostic strategies for the two most common CRDs. The detailed MEDLINE search strategy is shown in Supplementary Table 1.

Key websites (e.g. http://ghdx.healthdata.org) will be checked for eligible studies. We will follow up references of surveys with methodological papers, and will group papers from the same study (e.g. BOLD,^13^ FRESH AIR^4^). To avoid overlooking relevant local studies, we will not impose language restrictions; translations will be undertaken where possible.

### Study selection

Selection criteria and definitions are detailed in Table 1. Our focus is on surveys conducted in LMICs to study the prevalence of CRD (specifically asthma and COPD but also other CRDs) in randomly sampled community-based populations of adults. We anticipate that these will incorporate assessment of chronic respiratory symptoms and objective tests, measurement of risk factors for CRD, phenotypes of asthma/COPD or individual, healthcare or societal burden of CRD. We will undertake training at each step (100 randomly selected titles/abstracts or 50 full-text papers will be screened independently by D.A., N.S.H., H.P., E.M.K. and S.J.; they will be repeated until agreement exceeds 90%). After an initial shift to exclude obviously irrelevant titles (D.A., N.S.H.), two reviewers (D.A., N.S.H., S.C., S.-M.L. or E.A.B.) will screen the titles and abstracts, and then undertake full-text screening of potentially relevant papers, with disagreements resolved by discussion between researchers, arbitrated by a third reviewer (H.P., E.M.K. and S.J.) if necessary, and involving the wider research group to agree on conventions that operationalise the inclusion/exclusion criteria (see Table 1). The selection process will be undertaken using EndNote software (v9.0) and summarised using a PRISMA flow diagram.

**Table 1:** Inclusion and exclusion criteria

Criterion	Definition
Population	Populations of adults (typically of over 18 years, but we will accept different thresholds, for example, reflecting the age of the majority in different countries). Surveys that also screened children will be included if the procedure for adults is described
Screening procedure	Surveys employing random sampling with the aim of determining the prevalence of asthma,^50^ COPD^51^ or other CRD^8^ in the adult population. The survey procedures may include questionnaires, clinical examination, spirometry and/or other tests. We are also interested in the prevalence of chronic respiratory symptoms, and in surveys that detected phenotypes
Disease definitions	We will include studies that use definitions of CRD from globally recognised guidelines: asthma,^50^ COPD ^51^ or other CRD^8^
	We define ‘chronic’ respiratory symptoms as symptoms (such as cough, wheezing and shortness of breath) that have persisted for more than 3 months, or recurred in ‘attacks’
Burden of disease	We are interested in the population-level surveys of the burden of CRD; this includes symptom burden, use of healthcare resources or societal burden (e.g. absenteeism, loss of earnings)
Phenotypes	We are interested in population-level surveys that have attempted to detect phenotypes of asthma,^50^ COPD^51^ or the overlap between these conditions^53^ in the context of low-resource settings
Setting	Normally LMICs. Our, focus is surveys undertaken in low-resource contexts, which we anticipate will normally be countries classified by the Organisation for Economic Cooperation and Development as being ‘LMIC’ at the time of the survey. We may, however, include surveys developed in higher-income countries if the methodology employed was subsequently used in low-resource settings (e.g. BOLD)
Study designs	Prevalence surveys, including protocols for surveys

*COPD* chronic obstructive pulmonary disease, *CRD* chronic respiratory disease, *LMICs* low- and middle-income countries, *BOLD* Burden of Lung Disease

### Charting the data

Two reviewers (D.A., N.S.H., S.C., S.-M.L. or E.A.B.) will independently extract data onto a piloted customised data extraction form as follows: study metadata, country and populations, sampling strategy, CRD definitions, survey procedure (questionnaires used, spirometry and other measurements), risk factors measured, assessments of individual, societal and health service burden of disease and detection of phenotypes. Our research questions focus on identifying the process (rather than the outcomes) of undertaking surveys of CRD in LMICs, though we will note the prevalence of asthma, COPD and any other CRD identified in the included studies in order to inform future sample size calculations.

We will attempt to contact authors of the included papers for missing or unclear essential information; specifically, we will request copies of questionnaires or study procedures if they are not otherwise available. Multiple publications from the same study will be grouped, and draw on all the relevant publications, including methodological papers.^13^

### Collating, summarising and reporting the results

We will tabulate the procedures used and identify

- The strategies used to identify randomly sampled populations in LMICs
- The disease definitions used (which may vary over time)
- The questionnaires used and tests performed to detect asthma, COPD and/or other CRD
- Other variables for which information is collected in the included studies, in order to assess individual, societal and healthcare burden of CRD, and the risk factors for disease
- Whether recent surveys have addressed contemporary understanding of asthma/COPD phenotypes^41,50,51^ and the variables assessed to achieve this in low-resource settings

### Consultation

We will convene a workshop of the multidisciplinary research team with representatives from the RESPIRE partners (from Bangladesh, India, Malaysia, Pakistan and Edinburgh), at which the findings of the scoping review will be presented, discussed and interpreted. The conclusions will inform a future RESPIRE 4CCORD study, which will aim to estimate CRD burden in adults in Asian LMICs (initially Bangladesh, India, Malaysia and Pakistan).

### Dissemination

We will publish our findings in a peer-reviewed journal following the reporting standards for scoping reviews (PRISMA-ScR)^52^ and disseminate widely through our stakeholder groups and using innovative media approaches.

## Discussion

Assessment of the true prevalence for asthma, COPD and other CRDs underpins healthcare planning. Policymakers need assessments of the burden of disease if they are to plan and resource services that can accurately diagnose and effectively reduce the burden of CRD. Communication of the burden of CRD will raise awareness amongst stakeholders of the individual and societal impact of respiratory conditions and highlight the importance to communities of addressing potentially modifiable risk factors.

Limitations in currently available estimates of CRD burden in South Asian countries (e.g. relying on symptom questionnaires, or patient-reported diagnoses), hamper advocacy and are a barrier to development of healthcare service. Our scoping review will identify existing strategies used in surveys in LMICs, enabling future surveys to optimise their methodology and provide robust estimates of disease burden.

## Supplementary information

Supplementary Table 1.

## Supplementary information

**Supplementary information** accompanies the paper on the *npj Primary Care Respiratory Medicine* website (10.1038/s41533-019-0129-7).

## References

[1] Mathers CD, Loncar D (2006). Projections of global mortality and burden of disease from 2002 to 2030. PLoS Med..

[2] Adeloye D (2015). Global and regional estimates of COPD prevalence: systematic review and meta-analysis. J. Global Health.

[3] Salvi SS, Manap R, Beasley R (2012). Understanding the true burden of COPD: the epidemiological challenges. Prim. Care Respir. J..

[4] Van Gemert F (2015). Prevalence of chronic obstructive pulmonary disease and associated risk factors in Uganda (FRESH AIR Uganda): a prospective cross-sectional observational study. Lancet Global Health.

[5] WHO. *Monitoring the Building Blocks of Health Systems: A Handbook of Indicators and their Measurement Strategies* 1–92 (WHO, Geneva, 2010).

[6] Banda HT (2017). Community prevalence of chronic respiratory symptoms in rural Malawi: implications for policy. PLoS ONE.

[7] Lainez YB, Todd CS, Ahmadzai A, Doocy SC, Burnham G (2009). Prevalence of respiratory symptoms and cases suspicious for tuberculosis among public health clinic patients in Afghanistan, 2005–2006: perspectives on recognition and referral of tuberculosis cases. Trop. Med. Int. Health.

[8] Bousquet J, Dahl R, Khaltaev N (2006). GARD (Global Alliance against chronic Respiratory Diseases). Rev. Mal. Respir..

[9] Østergaard, M. S. et al. Recurrent lower respiratory illnesses among young children in rural Kyrgyzstan: overuse of antibiotics and possible under-diagnosis of asthma. A qualitative FRESH AIR study. *npj Prim. Care Respir. Med*. **28**, 13 (2018). 10.1038/s41533-018-0081-y PMC5893612 29636473

[10] Salvi S (2015). Symptoms and medical conditions in 204 912 patients visiting primary health-care practitioners in India: a 1-day point prevalence study (the POSEIDON study). Lancet Global Health.

[11] Bousquet J, Dahl R, Khaltaev N (2007). Global Alliance against Chronic Respiratory Diseases. Eur. J. Allergy Clin. Immunol..

[12] Contoli M, Papi A (2010). When asthma diagnosis becomes a challenge. Eur. Respir. J..

[13] Buist AS (2005). The Burden of Obstructive Lung Disease Initiative (BOLD): rationale and design. J. Chronic Obstr. Pulm. Dis..

[14] Anandan C, Nurmatov U, Van Schayck OCP, Sheikh A (2010). Is the prevalence of asthma declining? Systematic review of epidemiological studies. Eur. J. Allergy Clin. Immunol..

[15] Salvi S, Barnes PJ (2010). Is exposure to biomass smoke the biggest risk factor for COPD globally?. Chest J..

[16] Nasir KRN (2001). Epidemiology of cigarette smoking in Pakistan. Addiction.

[17] Nargis N (2015). Prevalence and patterns of tobacco use in Bangladesh from 2009 to 2012: evidence from International Tobacco Control (ITC) study. PLoS ONE.

[18] Lim HK (2013). Epidemiology of smoking among Malaysian adult males: prevalence and associated factors. BMC Public Health.

[19] Mishra S (2016). Trends in bidi and cigarette smoking in India from 1998 to 2015, by age, gender and education. BMJ Global Health.

[20] Lim S (2015). Impact of chronic obstructive pulmonary disease (COPD) in the Asia-Pacific region: the EPIC Asia population-based survey. Asia Pac. Fam. Med..

[21] Ahmed, N. *Study on Prevalence of Asthma and COPD at Dhaka City in Bangladesh*. Dissertation, East West Univ., Dhaka (2016).

[22] Alam DS, Chowdhury MA, Siddiquee AT, Ahmed S, Clemens JD (2015). Prevalence and determinants of chronic obstructive pulmonary disease (COPD) in Bangladesh. COPD J. Chronic Obstr. Pulm. Dis..

[23] WHO. *Global Tuberculosis Report 2017*. Licence: CC BY-NC-SA 3.0 IGO (World Health Organization, Geneva, 2017).

[24] Sultana T, Afzal A, Sultana S, Al-ghanim K, Shahid T (2017). Epidemiological estimates of respiratory diseases in the hospital population, Faisalabad, Pakistan. Braz. Arch. Biol. Technol..

[25] Subbarao P (2009). Asthma: epidemiology, etiology and risk factors. Can. Med. Assoc. J..

[26] Salvi S, Agrawal A (2012). India needs a National COPD Prevention and Control Programme. J. Assoc. Physicians India.

[27] Zhang Li (2017). Lung cancer in China: challenges and perspectives. J. Thorac. Oncol..

[28] Singh V, Sharma BB (2013). The ILD India Registry: a novel tool for epidemiological surveillance of interstitial lung disease in India. Indian J. Chest Dis. Allied Sci..

[29] Jahid H (2016). Interstitial lung disease (ILD) in India: Insights and lessons from the prospective, landmark ILD-India registry. Lung India.

[30] Dhar R (2015). Bronchiectasis in India: More Questions than Answers. RespiMirror.

[31] Chalmers JD (2017). The European Multicentre Bronchiectasis Audit and Research Collaboration (EMBARC): experiences from a successful ERS clinical research collaboration. Breathe.

[32] Mandal A, Kabra SK, Lodha R (2015). Cystic fibrosis in India: past, present and future. J. Pulm. Med. Respir. Res..

[33] Jindal SK (2010). Indian Study on Epidemiology of Asthma, Respiratory Symptoms and Chronic Bronchitis (INSEARCH). Rep. Indian Counc. Med. Res..

[34] Bishwajit G, Tang S, Yaya S, Feng Z (2017). Burden of asthma, dyspnea, and chronic cough in South Asia. Int. J. COPD.

[35] De Santi F (2017). Type 2 diabetes is associated with an increased prevalence of respiratory symptoms as compared to the general population. BMC Pulm. Med..

[36] Bartlett E, Parr J, Lindeboom W, Khanam A, Koehlmoos TP (2012). Sources and prevalence of self- reported asthma diagnoses in adults in urban and rural settings of Bangladesh. Global Public Health.

[37] Ahad A, Ming Khoo E (2017). Asthma control and care among Malaysian primary school children: a cross-sectional study. Asia Pac. J. Public Health.

[38] Chan YY (2015). Lifestyle, chronic diseases and self-rated health among Malaysian adults: results from the 2011 National Health and Morbidity Survey (NHMS). BMC Public Health.

[39] Loh LC (2016). Low prevalence of obstructive lung disease in a suburban population of Malaysia: a BOLD collaborative study. Respirology.

[40] Lai CKW (2009). Global variation in the prevalence and severity of asthma symptoms: phase three of the International Study of Asthma and Allergies in Childhood (ISAAC). Thorax.

[41] Pavord ID (2017). After asthma—redefining airways diseases. A Lancet commission. Lancet.

[42] Arksey H, O’Malley L (2005). Scoping studies: towards a methodological framework. Int. J. Soc. Res. Methodol..

[43] Chandler, J., Higgins, J. P. T., Deeks, J. J., Davenport, C. & Clarke, M. J. in *Cochrane Handbook for Systematic Reviews of Interventions Version 5.2.0* (eds Higgins, J. P. T., Churchill, R., Chandler, J. & Cumpston, M. S.) (Cochrane, https://community.cochrane.org/handbook-sri 2017). Available from Cochrane Community.

[44] Armstrong R, Hall BJ, Doyle J, Waters E (2011). “Scoping the scope” of a Cochrane review. J. Public Health (Bangk.)..

[45] Peters MDJ (2015). Guidance for conducting systematic scoping reviews. Int. J. Evid. Based Healthc..

[46] Levac D, Colquhoun H, O’Brien KK (2010). Scoping studies: advancing the methodology. Implement. Sci..

[47] Colquhoun HL (2014). Scoping reviews: time for clarity in definition, methods, and reporting. J. Clin. Epidemiol..

[48] Bateman ED (2008). Global strategy for asthma management and prevention: GINA executive summary. Eur. Respir. J..

[49] Pauwels RA, Buist AS, Calverley PMA, Jenkins CR, Hurd SS (2001). NHLBI/WHO Workshop Summary. Global Strategy for the Diagnosis, Management, and Prevention of Chronic Obstructive Pulmonary Disease NHLBI/WHO Global Initiative for Chronic Obstructive Lung Disease (GOLD) Workshop Summary. Am. J. Respir. Crit. Care Med..

[50] Global Initiative for Asthma. *Global Strategy for Asthma Management and Prevention.* www.ginasthma.org (2017).

[51] Global Initiative for Chronic Obstructive Lung Disease. Global Strategy for the Diagnosis, Management, and Prevention of Chronic Obstructive Pulmonary Disease Report. (2017).

[52] Tricco AC (2018). PRISMA Extension for Scoping Reviews (PRISMA-ScR): checklist and explanation. Ann. Intern. Med..

[53] Global Initiative for Asthma and Global Initiative for Chronic Obstructive Lung Disease Diagnosis of Diseases of Chronic Airflow Limitations: Asthma, COPD and Asthma-COPD Overlap Syndrome (ACOS). (2015).

