# Supplementary material for: Systematic scoping review protocol of methodologies of chronic respiratory disease surveys in low/middle-income countries
Source: NPJ Prim Care Respir Med. 2019 May 8;29:17. doi: 10.1038/s41533-019-0129-7 (PMC6506487; doi:10.1038/s41533-019-0129-7)
Supplement: Supplementary file 1 — Supplementary Table 1. [file 41533_2019_129_MOESM1_ESM.pdf]

Title:

Systematic scoping review protocol of methodologies of chronic respiratory disease surveys in low/middle income countries

Authors:

[#]Dhiraj Agarwal[1], [#]Nik Sherina Hanafi[2], Soumya Chippagiri[3], Evelyn A Brakema[4], Hilary Pinnock[5], Ee Ming Khoo[2], Aziz Sheikh A[5], Su-May Liew SM[2], Chiu-Wan Ng[2], Isaac R[3], Karuthan Chinna[2], Wong Li Ping[2], Norita Binti Hussein[2], *Sanjay Juvekar[1] and the RESPIRE Collaborators

Supplementary Table 1: Example of the search strategy developed for the MEDLINE database that will be adapted to search other databases.

**Ovid MEDLINE(R) 1995 to present with daily update—executed 03 Dec 2018**		
**#**	**Search Statement**	**Results**
**Concept 1: Chronic respiratory conditions**		
1	chronic respiratory disease*.ti,ab.	2555
2	(chronic obstructive pulmonary disease or obstructive airway* disease*).ti,ab	37579
3	((tuberculosis or TB) adj1 (post or history or anterior)).ti,ab.	326
4	Asthma.ti,ab.	122295
5	Asthma/	118555
6	chronic obstructive lung disease/	34328
7	exp interstitial lung disease	55562
**Concept 2: Outcome/ methods**		
8	(prevalence or incidence or epidemiol* or survey or rapid assessment or situation assessment or situational assessment or rar or cohort or surveillance or screening).ti,ab.	2217808
9	cohort analysis/ or mass screening/ or health survey	343972
10	epidemiology/ or cross-sectional study/ or exp disease surveillance/ or sentinel surveillance	297387
11	(cost or burden).ti,ab.	419205
**Concept 3 - LMIC**		
12	DEVELOPING COUNTRY/	71120
13	LOWEST INCOME GROUP/	0

14	MIDDLE INCOME GROUP/	0
15	RED CROSS/	2190
16	UNITED NATIONS/	6973
17	WORLD HEALTH ORGANIZATION/	31922
18	((developing or less* developed or under developed or underdeveloped or middle income or low* income or underserved or underserved or deprived or poor*) adj3 (countr* or nation* or population* or world)).ti,ab,kw.	79905
19	((developing or less* developed or under developed or underdeveloped or middle income or low* income) adj1 (economy or economies)).ti,ab,kw.	356
20	(low* adj1 (GDP or GNP or "gross domestic" or "gross national")).ti,ab,kw.	213
21	(low adj3 middle adj3 countr*).ti,ab,kw.	8264
22	(LMIC or LMICs or third world or LAMI country or LAMI countries).ti,ab,kw.	4724
23	(transitional country or transitional countries).ti,ab,kw.	131
24	(Africa or Asia or Caribbean or West Indies or South America or Latin America or Central America or Afghanistan or Albania or Algeria or Angola or Antigua or Barbuda or Argentina or Armenia or Armenian or Aruba or Azerbaijan or Bahrain or Bangladesh or Barbados or Benin or Byelarus or Byelorussian or Belarus or Belorussian or Belorussia or Belize or Bhutan or Bolivia or Bosnia or Herzegovina or Hercegovina or Botswana or Brasil or Brazil or Bulgaria or Burkina Faso or Burkina Fasso or Upper Volta or Burundi or Urundi or Cambodia or Khmer Republic or Kampuchea or Cameroon or Cameroons or Cameron or Camerons or Cape Verde or Central African Republic or Chad or Chile or China or Colombia or Comoros or Comoro Islands or Comores or Mayotte or Congo or Zaire or Costa Rica or Cote d'Ivoire or Ivory Coast or Croatia or Cuba or Cyprus or Czechoslovakia or Czech Republic or Slovakia or Slovak Republic or Djibouti or French Somaliland or Dominica or Dominican Republic or East Timor or East Timur or Timor Leste or Ecuador or Egypt or United Arab Republic or El Salvador or Eritrea or Estonia or Ethiopia or Fiji or Gabon or Gabonese Republic or Gambia or Gaza or Georgia or Georgian or Ghana or Gold Coast or Greece or Grenada or Guatemala or Guinea or Guam or Guiana or Guyana or Haiti or Honduras or Hungary or India or Maldives or Indonesia or Iran or Iraq or Isle of Man or Jamaica or Jordan or Kazakhstan or Kazakh or Kenya or Kiribati or Korea or Kosovo or Kyrgyzstan or Kirghizia or Kyrgyz Republic or Kirghiz or Kirgizstan or Lao PDR or Laos or Latvia or Lebanon or Lesotho or Basutoland or Liberia or Libya or Lithuania or Macedonia or Madagascar or Malagasy Republic or Malaysia or Malaya or Malay or Sabah or Sarawak or Malawi or Nyasaland or Mali or Malta or Marshall Islands or Mauritania or Mauritius or Agalega Islands or Mexico or Micronesia or Middle East or Moldova or Moldovia or Moldovian or Mongolia or Montenegro or Morocco or Ifni or Mozambique or Myanmar or Myanma or Burma or Namibia or Nepal or Netherlands Antilles or New	1583855

	Caledonia or Nicaragua or Niger or Nigeria or Northern Mariana Islands or Oman or Muscat or Pakistan or Palau or Palestine or Panama or Paraguay or Peru or Philippines or Philipines or Phillipines or Phillippines or Poland or Portugal or Puerto Rico or Romania or Rumania or Roumania or Russia or Russian or Rwanda or Ruanda or Saint Kitts or St Kitts or Nevis or Saint Lucia or St Lucia or Saint Vincent or St Vincent or Grenadines or Samoa or Samoan Islands or Navigator Island or Navigator Islands or Sao Tome or Saudi Arabia or Senegal or Serbia or Montenegro or Seychelles or Sierra Leone or Slovenia or Sri Lanka or Ceylon or Solomon Islands or Somalia or Sudan or Suriname or Surinam or Swaziland or Syria or Tajikistan or Tadzhikistan or Tadjikistan or Tadzhik or Tanzania or Thailand or Togo or Togolese Republic or Tonga or Trinidad or Tobago or Tunisia or Turkey or Turkmenistan or Turkmen or Uganda or Ukraine or Uruguay or USSR or Soviet Union or Union of Soviet Socialist Republics or Uzbekistan or Uzbek or Vanuatu or New Hebrides or Venezuela or Vietnam or Viet Nam or West Bank or Yemen or Yugoslavia or Zambia or Zimbabwe or Rhodesia).mp.	
25	exp AFRICAN/ or exp ASIAN/ or exp "CARIBBEAN (person)"/ or exp CENTRAL AMERICAN/ or exp EASTERN EUROPEAN/ or exp MELANESIAN/ or exp MICRONESIAN/ or exp POLYNESIAN/ or exp SOUTH AMERICAN/	65100
**Combined Searches**		
26	or/1-7 (247903)	247903
27	or/8-11 (2750986)	2750986
28	or/12-25 (1701254)	1701254
29	26 and 27 and 28 (7395)	7395
**Limits**		
30	limit 53 to yr="1995 -Current"	6762
31	(rat or rats or mouse or mice or monkey* or rabbit* or hamster* or bovine or sheep).mp.	3645043
32	animal/ or experimental animal/	6301604
33	31 or 32	6423247
34	30 not 33	6276
